# Systematic Study of the Content of Phytochemicals in Fresh and Fresh-Cut Vegetables

**DOI:** 10.3390/antiox4020345

**Published:** 2015-05-06

**Authors:** María Isabel Alarcón-Flores, Roberto Romero-González, José Luis Martínez Vidal, Antonia Garrido Frenich

**Affiliations:** Department of Chemistry and Physics (Analytical Chemistry Area), Research Centre for Agricultural and Food Biotechnology (BITAL), University of Almería, Agrifood Campus of International Excellence, ceiA3, E-04120 Almería, Spain; E-Mails: maf400@ual.es (M.I.A.F.); rromero@ual.es (R.R.G.); jlmartin@ual.es (J.L.M.V.)

**Keywords:** one-year monitoring, phytochemicals, vegetables, fruits, fresh, fresh-cut

## Abstract

Vegetables and fruits have beneficial properties for human health, because of the presence of phytochemicals, but their concentration can fluctuate throughout the year. A systematic study of the phytochemical content in tomato, eggplant, carrot, broccoli and grape (fresh and fresh-cut) has been performed at different seasons, using liquid chromatography coupled to triple quadrupole mass spectrometry. It was observed that phenolic acids (the predominant group in carrot, eggplant and tomato) were found at higher concentrations in fresh carrot than in fresh-cut carrot. However, in the case of eggplant, they were detected at a higher content in fresh-cut than in fresh samples. Regarding tomato, the differences in the content of phenolic acids between fresh and fresh-cut were lower than in other matrices, except in winter sampling, where this family was detected at the highest concentration in fresh tomato. In grape, the flavonols content (predominant group) was higher in fresh grape than in fresh-cut during all samplings. The content of glucosinolates was lower in fresh-cut broccoli than in fresh samples in winter and spring sampling, although this trend changes in summer and autumn. In summary, phytochemical concentration did show significant differences during one-year monitoring, and the families of phytochemicals presented different behaviors depending on the matrix studied.

## 1. Introduction

Vegetables and fruits are considered particularly protective for human health [1]. This characteristic is linked to the chemical composition of these foods, particularly to phytochemicals (known as bioactive compounds) [2], which include flavones, flavonols, isoflavones, phenolic acids and glucosinolates [3]. These phytochemicals seem to play a role against the development of different types of cancer and cardiovascular diseases, because these compounds could provide antioxidant capacity (AOC) [4], anti-inflammation properties [5,6], lipid profile modification [7,8] and antitumor effects [9,10]. In addition to these beneficial properties of phytochemicals in human health, they are responsible for the color, flavor and smell of fruits and vegetables [11].

Currently, all kinds of vegetables and fruits can be bought in all seasons, but their beneficial properties are not the same at every time of year. There are some studies that indicated a variation of phytochemical content in vegetables with the season, *i.e.*, glucosinolates in brassica vegetables [12,13], carotenoids in spinach, parsley and green onion [14], flavonols in tomato [15,16], onion and lettuce [16] and anthocyanidins in grape [17]. In general, most of these studies indicate that spring/summer season plants, which are grown at intermediate temperatures, high light intensity, longer days and dry conditions, have the highest content of phytochemicals. Conversely, the conditions in autumn/winter seasons are different (lower temperatures, lower light intensity, shorter days and higher water availability), and plants tend to have the lowest levels of these types of compounds. However, variation has also been observed throughout the harvest. For instance, Rosa and Rodrígues reported that some broccoli cultivars had higher glucosinolate concentrations in late season compared to the early season crop [18]. Moreover, tomato’s lycopene is inhibited at high temperatures (>30 °C) due to overheating [19]. Therefore, it can be observed that the seasons directly affected the concentration of phytochemicals in these matrices. Several factors, such as temperature, precipitation and radiation, can influence the accumulation of phytochemicals in vegetables and fruits. For instance, light exposure, especially ultraviolet-B rays, can provoke an increment in the content of these types of compounds [16]. Consequently, growing plants in greenhouses, which blocks ultraviolet light, reduces the flavonol content in plants. Vegetables grown outdoors contained four- to five-fold more flavonols than those cultivated in greenhouses [20]. Moreover, there is a trend indicating that higher flavonol levels were observed in warmer, sunnier climates than in cooler regions. Thus, Stewart *et al.* commented that growing conditions in Spain induce the accumulation of relatively high flavonols content in tomato fruits throughout most of the year [15].

These papers analyzed phytochemicals in vegetables and fruits from two [12,14] to four seasons [15,16], but few phytochemicals were determined. Therefore, the purpose of this work has been to provide a comprehensive view of the evolution of the content of more than 30 phytochemicals belonging to several families (phenolic acids, flavonols, flavones, glucosinolates and isoflavones) in fresh and fresh-cut products stored under modified atmosphere packaging (MAP), such as tomato, eggplant, grape, carrot and broccoli, during one year.

## 2. Experimental Section

### 2.1. Chemicals and Reagents

Commercial glucosinolate standards, such as progoitrin, gluconasturtiin and glucoraphanin, were supplied by PhytoLab GmbH & Co (Vestenbergsgreuth, Germany). Glucotropaeolin, glucoerucin and glucoiberin were purchased from Scharlab (Barcelona, Spain). Genistein, apigenin, quercetin, quercetin-3-*O-*glucoside, gallic acid, sulforaphane, ferulic acid, baicalein, gallic acid and caffeic acid were purchased from Sigma-Aldrich (Steinheim, Germany). Other standards, such as daidzein, glycitein, luteolin-4-*O-*glucoside, luteolin-7-*O-*glucoside, apigenin-7-*O-*neohesperoside, kaempferol, kaempferol-3-*O-*glucoside, kaempferol-3-*O-*rutinoside, luteolin, luteolin-6-*C-*glucoside, luteolin-8-*C-*glucoside, apigenin-7-*O-*glucoside, apigenin-6-*C-*glucoside, apigenin-8-*C-*glucoside, quercetin-3-*O-*ramnoside, quercetin-3-*O-*galactoside, quercetin-3-*O-*rutinoside, quercetin-3-*O-*ramnoside, quercetin-3-*O-*galactoside, isorhamnetin, isorhamnetin-3-*O-*rutinoside, isorhamnetin-3-*O-*glucoside, apigenin-7-*O-*rutinoside and tamarixetin, were purchased from Extrasynthese (Genay, France). Stock standard solutions of individual compounds (with concentrations between 200 and 300 mg/L) were prepared by exact weighing of the powder and dissolved in 10 mL of HPLC-grade methanol or in a mixture of methanol:water (50:50, v/v). Then, they were stored at −20 °C in dark bottles. A multi-compound working standard solution at a concentration of 5 mg/L of each compound was prepared by appropriate dilution of the stock solutions with methanol and stored in screw-capped glass tubes at −20 °C. The solutions were prepared every six months. Ultrapure water was obtained from a Milli-Q Gradient water system (Millipore, Bedford, MA, USA). Ammonium acetate was purchased from Panreac (Barcelona, Spain). Formic acid (purity > 98%), HPLC-grade methanol and dimethylsulfoxide were provided by Sigma (Madrid, Spain). Millex-GN nylon filters of 0.20 μm were provided by Millipore (Millipore, Carrightwohill, Ireland).

### 2.2. Apparatus and Software

Chromatographic analyses were carried out using an Agilent series 1290 RRLC instrument (Agilent, Santa Clara, CA, USA) equipped with a high-performance autosampler (G4226A), a binary pump (G4220A), a column compartment thermostat (G1316C) and an autosampler thermostat (G1330B). The system was coupled to an Agilent triple quadrupole mass spectrometer (6460A) with a Jet Stream ESI ion source (G1958-65138). For the chromatographic separation of the extracts, a Zorbax Eclipse Plus C18 column (100 mm × 2.1 mm, 1.8-μm particle size) from Agilent was used. Chromatographic conditions optimized in a previous work were used in this study [21].

An Agilent Mass Hunter Quantitative analyzer (Agilent Technologies, Inc.) was used for data acquisition and quantification of samples.

Statistical analysis (Analysis of Variance, ANOVA) was carried out with SPSS v21 (Armonk, New York, NY, USA).

Lyophilizer Alpha from Martin Christ (Osterode, Germany) was also used; an analytical balance AB204-S from Mettler Toledo (Greifensee, Switzerland), a Reax-2 rotary agitator from Heidolph (Schwabach, Germany) and vacuum pump from Vacuubrand (Wertheim, Germany) were also utilized.

### 2.3. Analysis of Samples

Samples were homogenized and were transferred to a Petri dish, and they were weighed and cooled to −18 °C. Then, all samples were processed according to the extraction method optimized in a previous work [21]. Briefly, 150 mg of lyophilized sample were weighed in a 15-mL polypropylene centrifuge tube and 3 mL of a mixture methanol:water (80:20, v/v) were added. The mixture was agitated for 30 min with a rotary shaker. After that, the extract was filtered and transferred into a vial containing mobile phase (50:50 v/v of Eluents A and B). A chromatographic method based on ultra-high performance liquid chromatography coupled to triple quadrupole mass spectrometry (UHPLC-QqQ-MS/MS) was used for the determination of the target compounds [21].

### 2.4. Samples

The sampling was performed at different seasons: February (winter), April (spring), July (summer) and October (autumn). For each sampling, fresh and fresh-cut vegetables and fruits were analyzed, and the same variety of each fruit and vegetable was evaluated.

Fresh tomato, broccoli, carrot, grape and eggplant samples was obtained from different supermarkets located in the province of Almería (southeast of Spain). On the other hand, fresh-cut products were obtained from Guzmán Gastronomía (Barcelona, Spain). In both cases, six samples of each matrix were analyzed. However, for fresh-cut carrot and fresh-cut eggplant, twelve samples per matrix were analyzed, bearing in mind that two types of cuts were evaluated for each matrix (grated and sliced for carrots and diced and sliced for eggplant).

Samples were chopped and homogenized at room temperature. After that, they were kept frozen at −18 °C until lyophilization.

## 3. Results and Discussion

### 3.1. Phytochemical Content in Tomato

**Figure 1:**
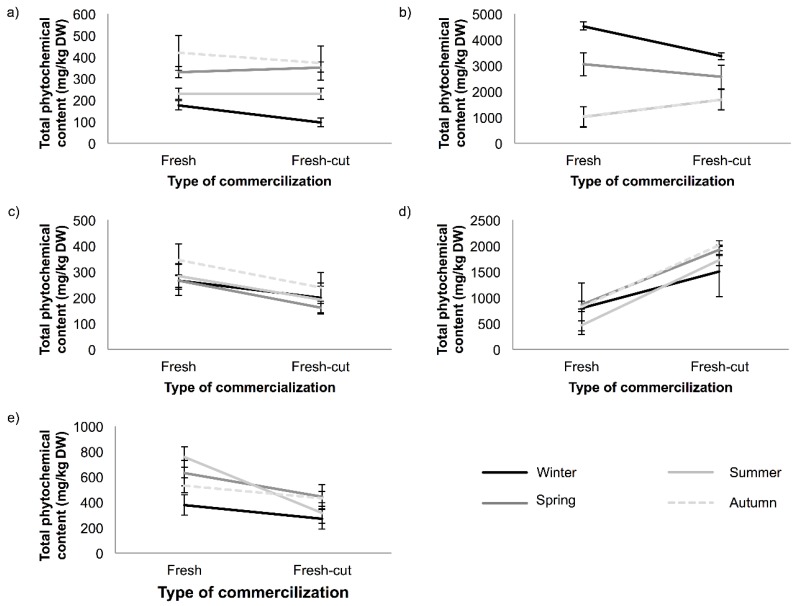
Total phytochemical content (mg/kg, dry weight (DW)) during the different seasons of the year in fresh and fresh-cut: (**a**) tomato; (**b**) broccoli; (**c**) grape; (**d**) eggplant; and (**e**) carrot.

The concentrations of phytochemicals in fresh and fresh-cut tomato are shown in Table 1. The effects of processing and sampling season were evaluated in tomato by two-way analysis of variance (ANOVA) study. When ANOVA was performed, it was observed that there is no statistical difference between fresh and fresh-cut samples during a year in the total content of phytochemicals, neither in phenolic acids nor flavones. However, there is a significant difference in the content of flavonols (*p* < 0.02), the content being lower in fresh-cut than in fresh tomato. In Figure 1a, it can be observed that the total content of phytochemicals in fresh-cut samples is lower in winter and autumn than in fresh samples, whereas in spring and summer, the total content is similar.

**Table 1:** Phytochemical content (expressed as mg/kg, dry weight (DW)) in fresh and fresh-cut in tomato, broccoli and grape in different seasons.

Matrix	Phytochemicals	Winter Sampling		Spring Sampling		Summer Sampling		Autumn Sampling	
		Fresh *	Fresh-Cut *	Fresh *	Fresh-Cut *	Fresh *	Fresh-Cut *	Fresh *	Fresh-Cut *
Tomato	Phenolic acids	102 (30) ^a^	54 (19) ^1^	225 (31) ^b^	294 (26) ^2^	97 (46)	107 (43)	148 (46)	127 (54)
	Flavones	29 (7) ^a^	21 (5) ^1^	37 (8) ^a^	24 (5) ^1^	39 (9) ^a^	62 (12) ^1^	104 (36) ^b^	113 (55) ^2^
	Flavonols	44 (20) ^a^	21 (11) ^1^	66 (16) ^a^	34 (17) ^1^	92 (17) ^a^	59 (12) ^1^	165 (29) ^b^	130 (94) ^2^
Broccoli	Phenolic acids	38 (11) ^a,c,d^	30 (8) ^1,3,4^	61 (16) ^b^	71 (26) ^2^	21 (9) ^a,c,d^	50 (12) ^1,3,4^	29 (12) ^a,c,d^	32 (15) ^1,3,4^
	Flavones	26 (4) ^a^	22 (10) ^1,2^	35 (5) ^b,c^	26 (10) ^1,2^	43 (3) ^c,d^	61 (27) ^3,4^	53 (6) ^d^	74 (19) ^3,4^
	Flavonols	13 (4) ^a,d^	5 (2) ^1,2^	35 (10) ^b,d^	15 (4) ^2,1^	58 (18) ^c^	41 (16) ^3,4^	32 (5) ^a,b,d^	43 (15) ^3,4^
	Glucosinolates	4449 (105) ^a^	3308 (198)	2929 (986) ^b^	2432 (945)	884 (237) ^c,d^	1517 (856)	919 (331) ^c,d^	1526 (877)
Grape	Phenolic acids	1 (1)	Not detected ^1,2^	3 (2)	2 (1)	1 (1)	1 (1)^12^	2 (1)	9 (8) ^3^
	Flavones	35 (7) ^a,b^	49 (22) ^1,2^	48 (8) ^a,b^	47 (7) ^1,2^	74 (4)	77 (31)	123 (66) ^c^	90 (21) ^3^
	Flavonols	230 (155)	149 (92)	213 (52)	112 (27)	207 (138)	114 (28)	220 (109)	139 (48)

* Mean value. The standard deviation is given in parenthesis (*n* = 6); mean values in italics indicate significant differences between fresh and fresh-cut matrices; in fresh samples, the mean values in a row with different superscript letters are significantly different (*p* < 0.05) using the Bonferroni test; in fresh-cut samples, the mean values in a row with different superscript numbers are significantly different (*p* < 0.05) using the Bonferroni test.

On the other hand, the total content of phytochemicals in each sampling was evaluated, being the difference only significant (*p* = 0.01) in the winter sampling (175 and 96 mg/kg dry weight (DW) for fresh and fresh-cut samples, respectively). Concerning the different families of phytochemicals evaluated, phenolic acids are the most abundant compounds in tomato; the level of this family fluctuated between fresh and fresh-cut in all samplings, but the difference was only significant (*p* = 0.02) in winter sampling.

**Figure 2:**
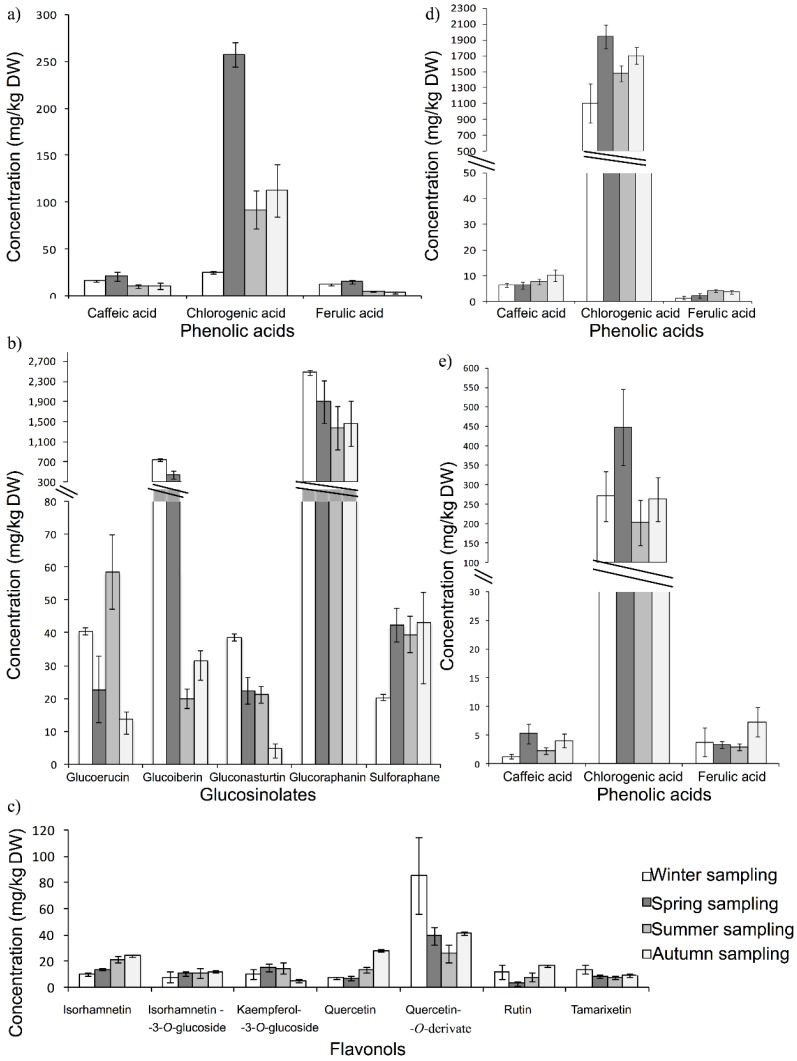
Concentration (mg/kg, dry weight (DW)) of the main family of phytochemicals in: (**a**) fresh-cut tomato; (**b**) fresh-cut broccoli; (**c**) fresh-cut grape; (**d**) fresh-cut eggplant; and (**e**) fresh-cut carrot during the different seasons of the year.

Total phytochemical content, as well as the different families studied were affected by season. Phenolic acids presented the highest concentration in spring sampling (225 and 294 mg/kg fresh and fresh-cut, respectively), although this difference was only significant when it was compared with winter sampling (102 and 54 mg/kg fresh and fresh-cut, respectively). Our data could not confirm that the amount of phenolic acids increases with increasing light intensity due to lower concentrations of phenolic acids (not significant) obtained in summer than in autumn and spring. This can be explained considering that high temperatures (>30 °C) can induce an overheating in tomatoes, which inhibits the biosynthesis of some compounds, as happens with lycopene in tomato [19]. On the other hand, growing conditions in Spain can lead to the accumulation of a relatively high phenolic acids content in tomato throughout most of the year. In relation to the levels of flavonols (165 and 130 mg/kg in fresh and fresh-cut samples, respectively) and flavones (104 and 113 mg/kg in fresh and fresh-cut samples, respectively) in autumn, they were significantly higher than in other samplings. However, Stewart *et al.* [15] and Raffo *et al.* [22] did not find any difference between tomatoes harvested in different seasons, observing that chlorogenic acid was the phenolic acid detected at the highest concentrations (from 26.7 to 54.4 mg/kg). In this study, chlorogenic acid was also the predominant phenolic acid in tomato in all samplings, as can be observed in Figure 2a, although the highest concentration was obtained in spring, which is in accordance with previous studies [22]. These results could indicate that chlorogenic acid content is affected by typical spring environmental conditions, although further studies should be performed in order to get definitive conclusions, because other factors, such as biotic and abiotic stress, can influence the final concentration.

### 3.2. Phytochemical Content in Broccoli

The concentration of the phytochemicals in different seasons in fresh and fresh-cut broccoli samples is shown in Table 1. In general, there was no significant difference in the total content of phytochemicals according to the type of commercialization (*p*-value = 0.83) throughout the year (Figure 1b). It can be observed that the total content of phytochemicals was very similar. When the content of the different families was evaluated, only flavonols showed significant differences between both types of products (*p* < 0.01), and their content is higher in fresh broccoli throughout the year.

The total content of phytochemicals was significantly affected by season, specifically between winter-spring sampling and summer-autumn sampling. This difference is attributed to the diminution of glucosinolates in summer and autumn, and these levels are higher in winter (4449 and 3308 mg/kg DW in fresh and fresh-cut samples, respectively) and spring (2929 and 2432 mg/kg DW in fresh and fresh-cut samples, respectively) than in the other seasons. This is in accordance with the results obtained from Henning *et al.*, who found higher levels of glucosinolates concentration in the spring season than in autumn [23]. However, Aires *et al.* [12] evaluated the content of glucosinolates in two different years, obtaining the highest concentration of this family in summer-winter in 2005–2006, whilst in 2006–2007, the highest concentration was obtained in spring-summer (3942 μmol/100 g DW). It was observed in Figure 2b that glucoraphanin was the predominant glucosinolate in broccoli in all samplings, as can be observed in previous studies [12], obtaining the highest concentration in the winter sampling (3737 mg/kg DW). In relation to the other families, significant differences were obtained, as can be observed in Table 1. The highest concentration of phenolic acids was obtained in spring (61 and 71 mg/kg DW in fresh and fresh-cut samples, respectively), which was statistically different than in the other seasons. The lowest concentration of flavonols was obtained in winter, which was statistically different than the content obtained during the rest of the samplings. The content of flavones was also affected by seasons, especially between the winter-spring sampling and the summer-autumn sampling, and flavones presented the highest concentration in the summer-autumn seasons.

### 3.3. Phytochemical Content in Grape

The concentration of phytochemicals in fresh and fresh-cut grape obtained during the four seasons evaluated is shown in Table 1. The results indicated that the total content of phytochemicals is not influenced by the type of commercialization. However, in all samplings, it can be noted that fresh grape has the highest concentration of phytochemicals (Figure 1c) ranging from 264 to 345 mg/kg DW, while in fresh-cut grape, the content ranged from 161 to 238 mg/kg DW. In relation to the families of compounds studied, no significant differences were observed among fresh and fresh-cut grapes.

Regarding the influence of the seasons, neither the total content of phenolic compounds nor the content of flavonols show significant differences. However, the content of flavones is significantly different depending on the season, because of the high concentration found in autumn (53 and 74 mg/kg DW for fresh and fresh-cut product, respectively).

In order to explain these results, the conclusions obtained by Ferrer-Gallego *et al.* must be considered [24]. They reported that there was a direct relationship between solar radiation exposure and concentration of phenolic compounds in grape, whereas there is an inverse relationship between accumulated rainfall and contents of phenolic compounds. Moreover, temperature does not have an influence on the content of flavonols in grape [25]. According to these results, it could be indicated that the grapes evaluated in this study have been cultivated in a place where the level of precipitation is low and solar radiation is high during the four seasons, because there were no significant differences in the content of flavonols (predominant group) and, consequently, in the total content of phenolic compounds. It can be observed in Figure 2c that quercetin 3-*O*-derivate was the most abundant flavonol in all samplings, although the highest concentration was obtained in the winter sampling (85.4 mg/kg DW in fresh-cut grape). In relation to other matrices, it can be noted that there is not a common phytochemical trend between different sampling times, indicating that each compound could be affected in a different way by the same environmental conditions.

### 3.4. Phytochemical Content in Eggplant

The obtained results showed a variation of the total content of phytochemicals between fresh and fresh-cut eggplant in all samplings (Figure 1d). This variation is mainly due to the fluctuations of phenolic acids among both types of products (fresh and fresh-cut), due to this family is the predominant group in eggplant. The highest concentration of phenolic acids was detected in fresh-cut eggplant, ranging from 1474 to 1875 mg/kg DW, whereas the content in fresh eggplant ranged from 385–799 mg/kg DW (Table 2). The higher levels in cut samples can be explained considering that during the preparation of the diced or sliced samples, the synthesis of the phenylpropanoid pathway is induced, and therefore, the content of phenolic compounds could increase [26].

It can be observed in Figure 2d that chlorogenic acid was the predominant phenolic acid in eggplant in all samplings, as can be observed in previous studies [27]. Flavones and flavonols also showed significant differences among fresh and fresh-cut samples throughout the year (*p* < 0.01 in both cases). Different types of cut in fresh-cut eggplant, diced and sliced, were analyzed, but in general, significant differences were not found. However, in a previous study [28], it was reported that there were significant differences in the content of phenolic acids between diced and sliced fresh-cut eggplant. This difference between both studies can be explained considering that in the current study, the effect of the type of presentation was evaluated throughout a year, whereas in the previous one, a single season only was evaluated.

When the ANOVA study was carried out to check if the sampling season affects the total content of phytochemicals in the assayed products, it was observed that this factor has no significant influence on the total content (*p* = 0.82). Bearing in mind the different families included in this study, it can be noted that the season significantly influenced the content of flavones and flavonols (*p* < 0.01), obtaining for both families the highest concentrations in autumn (62 and 87 mg/kg DW of flavones in fresh and fresh-cut samples, respectively). The content of flavonols was 53 and 48 mg/kg DW in fresh and fresh-cut samples, respectively, whereas the lowest level of flavones was obtained in spring (10 and 25 mg/kg in fresh and fresh-cut samples, respectively) and in winter for flavonols (24 and 10 mg/kg fresh and fresh-cut samples, respectively). However, no significant differences were observed for the content of phenolic acids (*p* = 0.86).

**Table 2:** Phytochemical content (expressed as mg/kg, dry weight (DW)) in fresh and fresh-cut in carrot and eggplant in different seasons.

Matrix	Phytochemicals	Winter Sampling		Spring Sampling		Summer Sampling		Autumn Sampling	
		Fresh *	Fresh-cut *	Fresh *	Fresh-cut *	Fresh *	Fresh-cut *	Fresh *	Fresh-cut *
Eggplant	Phenolic acids	733 (846)	1474 (649)	799 (137)	1867 (202)	385 (67)	1632 (197)	703 (241)	1875 (205)
	Flavones	30 (6) ^a,c^	30 (7) ^1,2^	10 (7) ^b^	25 (8) ^1,2^	39 (8) ^a,c^	56 (10) ^3^	62 (15) ^d^	87 (30) ^4^
	Flavonols	24 (14) ^a^	10 (6) ^1^	48 (22)	24 (12) ^2,3^	30 (8)	31 (6) ^2,3^	53 (9) ^b^	48 (16) ^4^
Carrot	Phenolic acids	340 (155)	236 (117)	564 (208)	378 (175)	652 (224)	239 (123)	407 (118)	249 (94)
	Flavones	26 (4) ^a^	24 (5) ^1,2^	46 (8) ^b^	45 (14) ^1–3^	69 (8) ^c,d^	54 (6) ^2,3^	87 (17) ^c,d^	114 (42) ^4^
	Flavonols	10 (5) ^a,b^	8 (4) ^1,2^	18 (3) ^a,b^	19 (5) ^1–3^	35 (9) ^c,d^	21 (3) ^2,3^	39 (7) ^c,d^	64 (20) ^4^

* Mean value. The standard deviation is given in parenthesis (*n* = 6 for fresh samples and *n* = 12 for fresh-cut samples); mean values in italics indicate significant differences between fresh and fresh-cut matrices; in fresh samples, the mean values in a row with different superscript letters are significantly different (*p* < 0.05) using the Bonferroni test; in fresh-cut samples, the mean values in a row with different superscript numbers are significantly different (*p* < 0.05) using the Bonferroni test.

### 3.5. Phytochemical Content in Carrot

The concentration of the total content of phytochemicals in fresh and fresh-cut carrots in different seasons is shown in Table 2. In relation to the content of total phytochemicals, it can be observed (Figure 1e) that there is a significant variation between fresh and fresh-cut carrot (*p*-value < 0.01), and phenolic acids are the predominant phytochemical family; the results for this family are shown in Figure 2e. Regarding the type of commercialization, significant effects (*p*-values < 0.01) were observed for the total content of phenolic acids, obtaining the highest values in fresh carrots, ranging from 340 to 652 mg/kg DW, whereas the content in fresh-cut carrots ranged from 236 to 378 mg/kg DW. These results are different from those obtained in a previous study [28], which reported that although differences between total phytochemicals and phenolic acids contents in fresh and fresh-cut carrots were observed, they were not significant. The discrepancy between both studies can be explained considering that in the current study, the effect of the type of commercialization was evaluated throughout a year, whereas in the previous study, a single season was only tested.

Season is a significant factor in the average content of total phenolic acids (*p* < 0.01) and, consequently, in the total content of phenolic compounds, the content in spring (564 mg/kg DW in fresh matrix) and summer (652 mg/kg DW in fresh matrix) samplings are significantly higher than in winter sampling (340 mg/kg DW in fresh carrot). In relation to the content of flavones and flavonols, it was high in autumn and low in winter, being these differences statistically significant (*p* < 0.01 in both cases), whereas in spring and summer, the detected concentrations were in the middle. These results are in agreement with those obtained by Leja *et al.* [29], who published that in the season of lower rainfall, higher amounts of phenolic compounds were accumulated in carrots. The found concentrations depended on the type of cultivar evaluated, and they ranged from 242 to 3115 mg/kg fresh weight.

Different types of cut in fresh-cut carrot, grated and sliced, were analyzed in all samplings, but no significant differences were observed in the total content of phenolic compounds (*p*-value = 0.16), as well as in the content of the different families in relation to the type of presentation of carrot throughout the year.

## 4. Conclusions

According to the data obtained in this work, it can be indicated that in relation to the matrix, the different families of phytochemicals have different behaviors when they are analyzed throughout a year. The several matrices harvested at different seasons of the year showed differences in the total content of phytochemicals, although these were not always significant. Autumn is the season with the highest concentration of phytochemicals in tomato, grape and eggplant, while the best season for broccoli was winter and spring, and summer for carrot. Although variations in total content or different families of phytochemicals according to the season have been observed, neither a clear seasonal trend, nor a correlation between phytochemicals content and mean solar radiation, nor average temperature was found. The total content of phytochemicals obtained was higher in fresh matrix than in fresh-cut matrix, as in carrot or grape. However, for eggplant, the highest concentration of phytochemicals was obtained in fresh-cut eggplant. In relation to broccoli, lower concentrations of phytochemicals in fresh-cut broccoli than in fresh broccoli were obtained in winter and spring.
